# Living with primary ciliary dyskinesia: a prospective qualitative study of knowledge sharing, symptom concealment, embarrassment, mistrust, and stigma

**DOI:** 10.1186/1471-2466-6-25

**Published:** 2006-10-13

**Authors:** Simon Whalley, IC McManus

**Affiliations:** 1 Department of Psychology, University College London, Gower Street, London WC1E 6BT, UK

## Abstract

**Background:**

Primary ciliary dyskinesia (PCD) is a chronic respiratory disease for which there is little psycho-social research and no qualitative studies of individuals living with the condition. A questionnaire-based survey in 2003 found evidence of stigmatisation in some individuals with PCD. Although the questionnaire had face and construct validity, stigmatisation was not cross-validated against interviews. The present study had the twin aims of carrying out a qualitative study of the adult patients living with PCD, and using a structured design to validate the questionnaire measure of stigma.

**Methods:**

Interviews were carried out with six pairs of individuals with PCD, matched for sex, situs, and age, one with a high stigma score in 2003 and the other with a low stigma score. Depth-qualitative interviews were conducted by one author to explore themes surrounding the psycho-social impact of PCD using a grounded theory analysis. The interviewer was blind to the stigma scores of participants, and after the qualitative analysis was completed, the interviewer made an assessment of which member of each pair seemed the more stigmatised, after which the code was broken.

**Results:**

Interviews revealed a number of themes, including other people's knowledge of PCD, the sharing of knowledge about PCD, the concealment of symptoms of PCD, embarrassment at symptoms, changes of behaviour in response to PCD, mistrust of medical care, in particular in relation to problems in diagnosis, a mistrust of general practitioners who were seen as poorly informed, and the importance of expert care at tertiary referral centres. Although stigmatisation as such was rarely mentioned directly by respondents, when the interviewer's judgement on level of stigmatisation was correlated with stigma scores from 2003, it was found that the more stigmatised member had been correctly identified in all six pairs (p = .016).

**Conclusion:**

Our results suggest that some people with PCD feel isolated through mistrust in medicine, and lack of knowledge surrounding PCD. Many responses to PCD can be explained in terms of stigmatisation, and in particular felt stigma. The correlation between questionnaire used several years previously, and the interviewer's judgements of stigmatisation suggest that the stigma questionnaire had both predictive validity and long-term stability. As in other chronic conditions, stigmatisation occurs only in some individuals with PCD, and the present study explores the basis of stigmatisation, and validate the questionnaire as a measure of difference in stigma.

## Background

Primary Ciliary Dyskinesia (PCD) (immotile cilia syndrome, Kartagener's or Siewert's Syndrome [1-3]), is a chronic respiratory disorder with a prevalence of about 1 in 15,000 – 30, 000 [4,5], usually inherited as an autosomal recessive [6,7]. Patients have abnormal or absent cilia, characterised by abnormal morphology of the 9 + 2 system of dynein arms [8]. Coordinated beating of respiratory tract cilia is disrupted, so that detritus from lungs and sinuses is not removed, resulting in chronic sinusitis, bronchiectasis and hearing problems. Dysfunctional beating of spermatozoa tails also causes infertility in males. Classically, Kartagener's Syndrome also shows left-right reversal of the viscera, *situs inversus totalis *(SI), in contrast to the normal arrangement (*situs solitus*, SS). The ciliary defect in PCD probably also occurs in the nodal cilia of the embryo determining *situs*. Disruption of normal right to left flow in PCD randomises *situs*, half of individuals showing SI and half SS [9-11,11]. Prognosis in PCD is good, with a normal life expectancy [12].

Diagnosis of PCD can be problematic, with wide variation in symptoms and scarcity of diagnostic facilities [4]. Age at presentation in one study varied from 4 months to 51 years [13]. Occasionally diagnosis is early, when associated with neonatal pneumonia [14]. There is currently no cure, treatment emphasising symptomatic relief, antibiotics for bronchitis, pneumonia and bronchiectasis, and regular physiotherapy, including postural drainage and chest percussion.

Despite relatively high numbers of patients with PCD – perhaps 3,000 in the UK and 12,000 in the United States – there are few studies of its psycho-social impact; apart from our own work [11,15], we know of only one personal report on the condition [16]. However, there are studies of psycho-social factors in related conditions, particularly cystic fibrosis (CF), a disease with parallels to PCD, including a genetic basis, chronic respiratory difficulties and fertility problems, although the far worse prognosis in CF limit the utility of the comparison [17,18]. In CF, diagnostic delay frustrated patients, both in childhood [19], and also, as is not uncommon in PCD, when diagnosis was delayed until adulthood [20,21], causing negative views of medicine, negative impacts on family life, less compliance with medication [22], and a need for information [21]. In childhood, CF resulted in problems in understanding the condition, in being "teased and picked on" [23] and telling others about the diagnosis [23,24]. However progressive deterioration in CF also produces specific problems, particularly in the transition to adulthood [25-29], a problematic time for all chronic chilhood conditions [30]. Although attitudes to chronic obstructive pulmonary disease (COPD) might be thought to overlap with PCD, the main problem in COPD is breathlessness [31], whereas cough predominates in PCD.

All chronic conditions put demands upon those with them. Qualitative research talks of the need to "achieve harmony with oneself" [32], and the difficulties of "hovering between suffering and enduring" [33]. Patients vary in their coping, with some identifying particular turning points and protective processes [34]. There is a recurrent realisation that doctors are ill-informed about the needs of chronic illness patients [35,36], and as patients with asthma put it, "We are the experts" [37], self-care being the norm [38]. PCD has the additional burden of a genetic component which adds to perceived problems of individuals and families [39]. Theoretical analyses of responses to chronic illness (such as the transactional model [30]), and models invoking loss and grief (see Sidell's review [40]), have little direct relevance to PCD, since although chronic, PCD is not acquired in adulthood but congenital, so there cannot be loss, grief or mourning as such, as nothing has been lost. Instead adjustment requires an acceptance of difference and deficit in childhood, which is perhaps best understood using the concept of stigma.

Elsewhere we have suggested that the impact of PCD upon psycho-social well-being [11], might be explained in terms of [15]. Of particular interest, is that since half of individuals with PCD have *situs inversus*, a physical but concealable bodily abnormality, decisions have to be made about revealing this atypicality to others [15]. The present study therefore included some subjects with *situs inversus*.

Erving Goffman's *Stigma: Notes on the Management of Spoiled Identity *[41] defined stigma as a mark that identifies the target individual as *spoiled *in some way (italics are used to indicate Goffman and others' technical terminology). This *spoiled identity*, through the *stigma mark*, devalues the individual in comparison to non-stigmatised individuals. Goffman identified three types of stigma: *abominations of the body *for physical problems such as facial blemishes, disability or stuttering; *blemishes of individual character *for character traits such as homosexuality, criminal behaviour, or radical political beliefs; and *tribal stigmas *such as race, religion, or nationality.

Individuals with PCD have no obviously visible stigmata (although the chronic cough is an audible sign). More relevant in Goffman's theoretical analysis is his differentiation of those whose stigma mark is instantly perceivable – *discredited individuals *– from those is not perceptible or known about – *discreditable individuals*. Although the latter have no problem of an obvious stigma mark, they still face problems of information management, deciding whether to divulge information, or to conceal the spoiled identity.

All recent work builds upon Goffman, although sociologists and social psychologists have different perspectives [42]. Jones *et al *[43] identified six separate components of stigma: *Concealability *– whether perception is necessary and inevitable; *Course of the mark *– the progressive development of the stigma mark; *Disruptiveness *– interference of social interactions; *Aesthetics *– the extent to which others are repulsed; *Origin *– perceived responsibility for the stigma mark; and *Peril *– possible dangers posed by the stigmatised person. A separate theoretical distinction, developed in a study of epilepsy by Scambler and Hopkins [44], differentiated public acts and outcomes of *enacted stigma*, from the more complex, personal shame experienced in *felt stigma*. Other theoretical facets include aspects of stigma and stereotypes [45], prejudice [46], embarrassment [47], and social control [48].

Measurement of stigma has proved problematic, particularly using questionnaires, which have focussed on topics as family relations, social interaction, and self-esteem [49], or retreat, self-esteem, rejection, concealment, and composure [50]. A review by van Brakel [51] emphasised that "the consequences of stigma are remarkably similar in different health conditions", implying a generic instrument should be possible, and justifying sharing questionnaire items across conditions, as we did in our previous study [15], developing a scale (see Table 1), which adapted items from a scale developed for people with Parkinson's disease [52]. Factor analysis showed a single underlying dimension. Path analysis found that neither physical symptoms nor the presence of SI related to perceived stigmatisation in PCD, whereas personality, mental health status, and social impact did predict stigma levels.

Although our previous study [15] had developed a stigma questionnaire with face and construct validity, there was no direct validation. The present study therefore had two separate aims: to use qualitative methods to explore personal responses to PCD, with the particular possibility of stigma; and to use a quasi-experimental design for directly validating the stigma questionnaire.

A literature search found no qualitative studies of PCD, and so we used a grounded theory approach. Grounded theory [53], which comes in several 'flavours' [54], is useful for capturing patient perspectives and understanding the meanings people attach to their social world. In general, grounded theory should not involve *a priori *theoretical influences, allowing specific themes, in our case on PCD in general and the possibility of stigma, to emerge through the data collection and analysis process [54].

The present qualitative study followed-up participants from the original quantitative study, in a purposive, matched-pairs design, one member of each pair in 2003 having a high stigma score and the other a low score. A key feature of the study is that all interviews, transcriptions and interpretations of qualitative data were made by SW, who did not have access to the original questionnaire data, whereas the pairs of interviewees were chosen by ICM, who did not take part in the grounded theory analysis. Only when grounded theory analysis was complete did SW rate the extent of stigma in the pair members, the similarity of those ratings to the original stigma scores assessing both the validity and long-term reliability and stability of the questionnaire.

## Methods

### Sampling and recruitment

In January 2003, 160 members of the UK Primary Ciliary Dyskinesia Support Group with diagnosed PCD were sent a 16-page questionnaire covering a wide range of topics, including mental and physical health status [15]. The 93 completed questionnaires form the primary sampling frame for this study.

**Figure 1:**
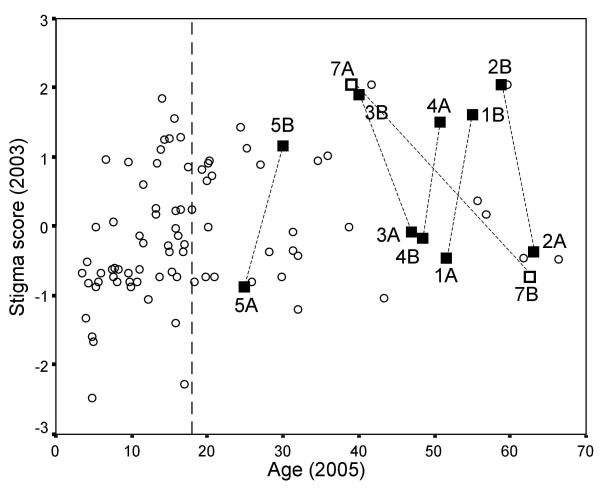
Questionnaire-based stigma scores in relation to age, for all participants in the 2003 questionnaire. Participants taking part in the current study are indicated by squares, with open squares for males and solid squares for females, and their pair membership shown.

ICM was responsible for choosing participants. Figure 1 shows stigma scores in 2003 in relation to participants' age in 2005, when this study was carried out. Individuals under the age of 18 were excluded for ethical reasons, and for practical reasons individuals included lived within 250 kms of London. Pairs were identified with one 'high' stigma scorer (≥ 1) and one 'low' stigma scorer (≤ 0), each with the same situs and sex, and where possible, ages within ten years of one another. SW contacted participants by letter and then phone. Figure 1 shows age and stigma scores for the eventual six pairs. The database contained more females than males, and matching male pairs was difficult. Ill health caused the late withdrawal of one male participant and when another match was found the ages were somewhat discrepant (42 and 65). Although the original study design looked for seven pairs (fourteen participants), pair six was eventually dropped because a further match could not be found after one participant dropped out. The results here describe six pairs (labelled 1,2,3,4,5 and 7), aged from 27 to 65 (mean 49.8), and three pairs showing SI. Table 2 summarises the demography, and Table 3 shows 2003 responses to the St George's Hospital Respiratory Questionnaire [55] and the derived scales of the SF-36 health questionnaire [56,57].

### Interviews

Using a grounded theory analytical approach (see below), depth-qualitative interviews were conducted to allow for a full exploration of themes surrounding the psycho-social impact of PCD. All interviews were conducted by a single interviewer (SW) who was blinded to the original stigma scores until full coding and analysis had been completed. A prompt card was used by the interviewer during each interview to ensure that key areas were covered. Interviews were conducted between July 2005 and January 2006 (hereafter '2005'), lasted 40 to 60 minutes and were recorded and transcribed for analysis. Eleven interviews were conducted in participants' homes, and one in the UCL Psychology Department.

Three pilot interviews ensured that SW understood the key symptoms, treatments and experiences of people with PCD. While the first two pilot interviews focussed on symptoms and diagnosis, the final pilot interview included more in-depth psycho-social issues concerning PCD, and this interview was included in the final analysis (participant 3A).

### Grounded theory analysis

To ensure data collection was truly grounded, analysis was carried out between interviews, as well as after all interviews were completed [58]. Initial themes under investigation included diagnosis, symptoms and social perspectives surrounding PCD, including the possibility of stigma. To ensure theories and concepts were grounded in collected data [54] the focus of the interviews changed as themes emerged during the data collection phase, while other themes were replaced when saturation was reached and new information was not forthcoming. Before each interview the previous interview was fully transcribed and loosely open-coded, with emerging themes compared and contrasted to previous interview data. A field journal was updated following each interview to capture any new themes emerging, and where relevant these themes guided subsequent interviews and analysis.

After data collection the complete transcriptions were comprehensively open-coded to formulate a list of key themes that may affect levels of stigma in people living with PCD. Data were initially grouped within five themes: knowledge of PCD; management of PCD; the 'self'; living with PCD; and support. Following subsequent coding and analysis, key themes were finally grouped within three main categories: knowledge surrounding PCD; concealment of PCD; and mistrust of medical care. Anonymised participants' extracts are used in the qualitative results section of this report, based information on each participant being presented in the form [2B: 61F, SI, high], indicating [Pair identifier; Age and sex, situs, stigma score].

### Stigma ratings

When qualitative data had been coded and analysed, SW rated each participant on a four-point scale for perceived stigma (1: no perceived stigma, through 4: high perceived stigma). These ratings, which were blind questionnaire stigma scores, were based upon an informal subjective analysis of psycho-social themes within the qualitative data, including self-reported symptom concealment, trust in medicine, and current and past social support. For clarity, quantitative, questionnaire-based stigma assessments in 2003 are referred to as *stigma scores*, whereas interviewer assessments of stigma in 2005 are referred to as *stigma ratings*.

### Un-blinding and comparison of stigma scores

When SW had made his stigma ratings, ICM revealed the original questionnaire-based stigma scores, and scores and ratings compared. Although our original study design allowed SW to re-analyse transcripts in the light of discrepancies between stigma scores and ratings, that in fact was not required.

### Ethics and confidentiality

Ethical approval was granted by the University College London Hospital Local Research Ethics Committee. Participants provided informed consent, were fully briefed on the study, told they could withdraw at any time, and that interviews would be recorded and transcribed and were fully confidential. Participants received no payment for taking part, but were thanked for their time.

## Results

### i) Qualitative findings

#### Knowledge of PCD

##### Other people's knowledge of PCD

When asked about other people's knowledge of PCD, all participants said other people knew little or nothing about PCD, although feelings were mixed about this. Most participants accepted that the low prevalence of PCD meant there would be a paucity of public knowledge, but nevertheless some participants spoke of frustration that lack of knowledge led to lack of support and understanding:

"...it's frustrating when you need the treatment and if you're feeling rough people don't understand sometimes, things like that but it is how it is and you have to deal with it the best way you can, don't you." [1B: 57F, SI, high]

"I don't know anyone who's got the condition, I don't know anyone who's got a chest complaint even, not a chronic one, apart from a few touches of asthma. I suppose it's horrible really but they make an awful fuss and, but no, it's lonely, if you have cancer [...] most people know someone else and you have a sort of support through that but this is an odd one, chronic chest condition..." [2B: 61F, SI, high]

However one participant spoke of an advantage of having a rare condition such as PCD:

"Well, other people are very interested because it's quite an interesting condition to have, especially being so rare." [1A, 54F, SI, low]

##### Sharing knowledge of PCD

Some participants used cystic fibrosis to anchor understanding about PCD:

"And you can always fall back on the well it's a disease, the symptoms are not dissimilar to cystic fibrosis because everybody's heard of CF these days." [1A: 54F, SI, low].

There was also a link between educating others about PCD and disclosing details of their own condition:

"...so if I tell someone I've got it I have to explain what it is." [5A: 27F, SS, low].

This produced a dilemma for some participants about whether to disclose, and how much information should be given. One participant's example of disclosure seemed open and uncensored by containing references to possible stigmatising signs:

"...normally say, you know, I've got, I've got this thing called PCD and it's, basically I go you know the little hairs in your body or cilia that waft along in your bronchial tubes and your nasal passages and they go no (laughs). Well anyway, your tubes are lined with microscopic hairs and they waft all the muck out and keep your lungs clear and I say well mine don't work properly and so, you know, I get all the muck and obviously it's not very good for the lungs, or lung, and, yeah, but just explain it layman's terms." [4B: 51F, SS, low]

Another participant seemed comfortable with people finding out about all symptoms of PCD either via the internet or through personal disclosure (interviewer comments in italics):

Interviewer "And do you actively kind of educate people about it [PCD] sometimes?

"Yeah, I do. If I, I mean obviously I would make sure that I explained it so that they know that they exist and [...] if they asked me questions then I would try and tell them and I might refer them to the website or something." [5A: 27F, SS, low]

However, most participants conducted varying levels of information management. Information regarding PCD often seemed to be self-censored by avoiding revealing outwardly stigmatizing signs, such as a chronic cough or excessive nose blowing:

Interviewer: "What are the main points that you might tell them about?

"Oh, about the immotile cilia, I mean everybody I've come across knows that my [...] heart's in the right and liver's on the left so that's fairly basic and everybody's interested in that but I won't go into details about productive cough because that's not something people want to know about." [1A: 54F, SI, low]

"I might tell them about the.., I might tell them the, you know, name of it. I just hope they won't look it up (laughs). But I don't tell them the actual symptoms. I might say about the cilia not working, but I don't actually say the consequences of that." [1B: 57F, SI, high]

"I would, well if I say anything I would just say it affects the cilia that are in your lungs so that they don't beat properly and the, as a result you get lung damage." [4A: 53F, SS, high]

There were differences in reasons for disclosing information about the condition, one participant talking of a benefit in being open and educating others about their PCD:

"I think it's important they understood, and the more you tell people about it, the more you feel you know about it yourself." [7B: 65M, SS, low]

However, there were also situations where there was a felt pressure to disclose:

"...because I've told my neighbours mainly because I think some of them wonder why I haven't had any children." [2A: 65F, SI, low]

"I've been going to yoga and again I've got into my loop of trying to hide it and quite recently I did tell the teacher in detail, not in detail in two sentences and what had happened was they'd had a stand-in teacher who had made a spectacle of me saying oh, why aren't you doing that and why can't you do that and I said well I can't do it because it's too strenuous and I thought oh I'm just going to have to tell them really." [2B: 61F, SI, high]

"I don't go out my way to tell people I've actually got anything wrong with me but obviously people I might be working close with, you know, if I have had a spell in hospital obviously then they're going to want to know why..." [4B: 51F, SS, low]

Some participants had at some point avoided disclosure, or been discouraged to do so, and this was particularly salient at school:

"...but, yeah, at school I did [have time off ill], you know [...] at senior school I used to, it was an age thing where I didn't want to admit to my friends that I had anything wrong with me and I'd say oh I've got a cold, I can't get rid of it, and I'd never talk about it so consequently I just carried on doing the same kind of stuff that they did. And I just couldn't keep up." [3B: 42F, SI, high]

"... I know at one point the headmistress of the grammar school I was at wrote to my mum and said why was I having so much time off and she wrote back and said I had bad periods which wasn't true at all so there was this sort of hiding it which I don't, I think there's a bit of a fine divide between cosseting somebody too much and letting people understand that you actually can't do things for a reason." [2B: 61F, SI, high]

#### Concealing PCD

##### Hiding the symptoms of PCD

Of the twelve participants, nine had at some time attempted to conceal symptoms of PCD. Of the remaining three, two were in the low stigma group (1A and 7B) and one in the high stigma group (2B). 1A and 2B said it was simply too hard to conceal a cough (see below), while 7B had concealed symptoms only as a child. Other participants also tried to hide symptoms when younger:

Interviewer: "Can you remember what you might have done at the time, if you needed to cough?

"I would go out. I would try and pretend that you know, I would try and hold back from, you know, where you try and hold back from coughing and you try not to cough and then it gets worse and it escalates, and then it all explodes out, [...] that sort of thing would happen. Or I would go out the room or go away and cough." [3B: 42F, SI, high]

"...so I just used to hold my breath, try and hold my breath in class and watch the clock. Because obviously if I breathed too quickly or too deeply I might start coughing so I used to sort of sit there and (laughs) just praying to god that I would reach the end of the lesson without coughing, and then go out to the loo." [1B: 57F, SI, high]

Participants particularly attempted to hide coughing and nose-blowing. As well as concealing symptoms at school, some participants also described current attempts at hiding outward signs of PCD:

"I do things like if we're in a restaurant or something I'll go to the loo and cough in there except sometimes the friend will come with me and I'll think oh no, so that I go later. And I do thing like that just to try and deal with it privately as possible." [4A: 53F, SS, high]

"I'll blow my nose in the toilets generally if I'm out anywhere I'll blow my nose in the toilet so I cough in the toilets, I'll go anywhere other than be around other people, because I know people don't like to hear it and, you know, so I, yeah, I've tried to disguise and hide and quiet it, and almost always, almost always." [7A: 42M, SS, high]

One participant described how, when younger, she hid her cough for practical reasons:

"probably from my mum to avoid going to the doctors or to avoid having physio..." [5A: 27F, SS, low]

However, two participants were pragmatic regarding their symptoms when asked if they had ever attempted to hide their PCD:

"...no I don't think so...well no, it's very hard to suppress a cough anyway. And if you've got a cough and a wheeze you've got a cough and a wheeze, there's not much you can do to suppress that". [1A: 54F, SI, low]

"Bit difficult (laughs). I can't. Because if you've got a cough, you've got to cough. If you've got to blow your nose, you've got to blow your nose so I can't. No. I feel sorry for people hearing me sometimes but most people have been very good at not noticing it." [2B: 61F, SI, high]

##### Embarrassment at PCD symptoms

One participant who concealed her symptoms summed up her feelings when asked how PCD affected her at school and work:

"Oh, it made me feel like a dirty old man, quite honestly. I think that says it all honestly." [1B: 57F, SI, high]

The reasons of some participants for concealing symptoms suggested felt stigma, with excessive coughing seeming to be the main concern:

"I suppose it's not very...actually coughing up mucus isn't a very nice thing. It's not, it's quite a sort of...frowned on in society kind of thing isn't it so I kind of, yeah, I don't think it's very nice, sort of, to do it in front of people, so, yeah, I don't know that I'm scared about people finding out particularly but, yeah, just...yeah, don't know, not really thought about why, you know, why. But isn't just that it, yeah, doesn't seem like a very nice sort of social thing to do and that, maybe would think differently of you, or kind of, yeah, look at you differently..." [5B: 32F, SS, high]

"So as a child my nose ran constantly, I would take to school a pile of large handkerchiefs, and, you know, I wanted to cough all the time basically, yes, but I tried to suppress it because I was very embarrassed by it." [1B: 57F, SI, high]

Interviewer: "And can you remember why you first started doing it [hiding the symptoms]?

"Because people used to, sort of, because as you cough people sort of give you a funny look and the eyebrows wrinkle up and I knew that they found it unpleasant and certainly as far as dating and things like that, it was something I had to be, I, it's quite something for someone to get used to really..." [7A: 42M, SS, high]

##### Behaviour change

Participants concealing symptoms also described changing behaviour in order to conceal, as when a participant missed postural drainage sessions:

"...this is probably going to sound ridiculous but like, if we go to stay with friends for a weekend I don't do it [postural drainage] because if I could do it silently I would, but I can't..." [4A: 53F, SS, high]

Another participant talked of problems when younger and on holiday:

Interviewer: "You mentioned holidays earlier on, but why do they present a problem or a fear for you?

"Well, because of the, you know, postural, well, I didn't do postural drainage in those days, but I went on a camping holiday with friends and parents. It was a nightmare because obviously then I didn't know how to deal with it I suppose I was about, don't know, thirteen or something, and, you know, trying to cough in a tent (laughs). It was so embarrassing. So when they asked me next year, I didn't go on holiday. That was it really. And I didn't even go on days out like school outings. I went once, you know, and, you know, it just wasn't worth it. So, I got used to not going on holidays." [1B: 57F, SI, high]

In contrast, one participant who had never concealed symptoms was open about her condition and treatment:

"I think I've become, I've become more aware of it [treatment]. I'm more aware that exercise isn't an option, or it isn't shall I or shan't I. I'm beginning to be aware that, especially since I started having bone density scans [for osteoporosis secondary to earlier steroid treatment], that, that this, this is serious stuff, and I know that if I do the exercise and I'll be half-way home and I'll cough and I'll that blob of phlegm that's blocking some airway somewhere, well that's good." [1A: 54F, SI, low]

Whilst some participants' behaviour had been largely unaffected by PCD, others' behaviour was highly disrupted. Some participants reported career changes to avoid poor air conditions or because of enacted or felt stigma. One participant who appeared unaffected by felt stigma had nevertheless left a job because of poor conditions:

"...when I first left school I worked at [a car company] showroom not far from here and I think I'd only been there six months, the trouble was I worked in a small office with a heavy smoker and within about six months I did get pneumonia so I got pneumonia when I was seventeen, I was quite poorly with that, then I left there because I'd decided maybe heavy smoking wasn't good for me..." [4B: 51F, SS, low]

##### Situs inversus

Although the situs inversus shown by many people with PCD might seem to have the potential for being stigmatising, none of the participants saw it in that way, merely seeing it as being interesting:

"*Interviewer: Did you feel different in any way at all at school?*

Er hmm (long pause) not until I suppose in my teens when I I knew it was the wrong way round inside but that was quite nice because that felt quite special.

Interviewer: Right

(unintelligible) James Bond's heart is the other way round as well you know you sort of think 'wooh'. [...] So I was just able to say 'Oh I'm the wrong way round inside.' Not a big attraction really to any people." [3A: 49F, SI, low]

#### Mistrust of medical care

##### Uncertainty of diagnosis

Many participants experienced uncertainty over diagnosis, with age at diagnosis ranging from 10 to 58, and most participants experiencing bronchiectasis or other problems before a diagnosis was reached. Failure to diagnose PCD early left many participants with a mistrust of medical care:

"I had so many conflicting things that I was told, and then when I was nineteen I was told that by the time I was thirty I would be permanently in a wheelchair and on oxygen, and I mean at the time I was quite upset by it, and so I suppose I've learnt to take a big pinch of salt with what any medical person tells me." [4A: 53F, SS, high]

"What I will say if I suppose, I've only got really one thing to say, I think [...] doctors don't always like to admit that they don't know something, do they. And I think when I was younger, you know, if they'd said they didn't know [...] and perhaps I'd have more help in how to manage it, even if they didn't know what it was. That would have been really helpful." [1B: 57F, SI, high]

Interviewer: "When were you first diagnosed with PCD?

"I don't know. One of these things that I've drifted into an awareness of it. I think for most of my childhood, teenage years, young adult it was labelled as bronchial asthma. I rather have a feeling that they didn't think it was bronchial asthma but they put a label on it...for the sake of putting a label on it." [1A: 54F, SI, low]

Older participants experienced greater problems with uncertainty over diagnosis – including partial lung removal before a PCD diagnosis was made – but agreed that medical knowledge seemed to have improved. However, there was a sense of abandonment for one participant:

"There's really a change of life from being a child but I think now, nowadays things are very different, children are involved in their treatment and understand their conditions so much better, partly the organisation's [PCD Support Group] done a lot for that. But apart from us older ones then no I don't think things will change very much at all." [2B: 61F, SI, high]

While most participants spoke of the problems due to late diagnosis, some, despite such problems, were still trusting of doctors, as in a participant who had had several antral punctures to improve sinus drainage:

"It wasn't an enjoyable process but I think well, you know, because you accept whatever's happening really, don't you (laughs).

Interviewer: "Where do you think that acceptance came from?

"Faith I suppose in the doctor. I thought well he wouldn't be doing all this if there wasn't a reason for it. And mum wouldn't have brought me here for no reason. I suppose it was that really." [2A: 65F, SI, low]

##### Mistrust of GPs

Some participants described problems with general practitioners (GPs), particularly when needing antibiotics for chest infections, with a sense of isolation due to poor communication between GP and specialist:

"...the doctors here weren't used to that strength of antibiotics and they didn't want to prescribe them so at one point I had to write to the doctor here explain what antibiotics the Brompton were telling me and write to Doctor [...] at the Brompton can you write to my doctor to tell him. Which he did." [3A: 49F, SI, low]

"What they tend to do is give you Amoxicillin. If that doesn't work they might try something else but there's very little knowledge of PCD, certainly within my doctor's surgery then I, every time I see the doctor they're sort of a bit betwixt and between and they've seen, I've got notes like this wad of them and I feel that maybe that is an area where it could, maybe more liaising with Brompton and the doctors so they're a bit more aware rather than me having to explain it every time that I go to them and I'd ring up the doctor's surgery and they say can I make an appointment for two weeks' time and I say well really need to see them soon because damages your lungs each time." [7A: 42M, SS, high]

However, some participants, also knew how to obtain access to the necessary antibiotics:

"He's [GP] OK. I basically go in there and he'll say what do you need and I'll tell him what I need and then I'll walk out again (laughs)". [3B: 42F, SI, high]

"...it's all being proactive rather than reactive about the, the use of these things [antibiotics], just in case, you know, with doctors you have to wait five days for a doctor's appointment, or you can sit and wait a few hours in the day, I mean I don't feel like doing that. So I'm proactive in that way and of course I'm very aware of what I've got and how it works now". [7B: 65M, SS, low]

Whilst the interviews suggested mistrust because of early problems with misdiagnosis, the Royal Brompton Hospital, the tertiary referral centre where most participants received their diagnosis, was universally praised, with comments about participants' happiness at finally people who understood their condition:

Interviewer: "How did you feel being at the Brompton, how was it?

"It was great. It was almost like for the first time somebody's listening to me and doing something that I needed doing..." [3B: 42F, SI, high]

"I suffered with heartburn for a very long time and, years, and I went to Brompton and they said you get heart burn don't you and I like yeah he said give you some tablets for that and took the tablets for two weeks and then that cleared my heartburn up but I'd had that for years beforehand you see". [7A: 42M, SS, low]

#### Stigma questionnaire scale validation

Stigma ratings from 2005 were compared with stigma scores from 2003 (see Table 2), and showed complete concordance, all participants with higher stigma scores in 2003 having higher stigma ratings in 2005 (Table 2). Calculating statistical significance is straightforward. For a particular pair SW had a 50% chance of guessing which participant had the higher stigma score. The chance probability of six correct guesses is therefore .5 × .5 × .5 × .5 × .5 × .5 = .5^6 ^= 1/64 = 0.01562, i.e. p < .05. In contrast, paired t-tests on five measures of physical and mental health (Table 3), found no significant differences, suggesting SW's judgements were specific, and not secondary to perceived health status.

## Discussion

This qualitative study of PCD has identified a number of important themes, including knowledge sharing, symptom concealment, embarrassment, and mistrust of doctors and medical care. The unusual methodology, combining a questionnaire study and a focussed qualitative analysis of matched sub-groups differentiated maximally on stigma scores, is an example of the hybrid technique of "matching and thick description" [59].

### Stigma

A particular interest concerns stigma, since there is no specific stereotype for PCD, and indeed no participant could think of one. Nevertheless, nine of twelve participants concealed their condition at some point, suggesting that although the condition itself is not stigmatising, its symptoms may be, as Scambler suggests [44,60], particularly given the negative status of prominent chronic productive cough [61]. That conclusion is particularly supported in PCD, since only a random 50% of patients show the statistically rare sign of *situs inversus*, and yet they seem to show no higher levels of stigma than those with *situs solitus*. An unusual feature of PCD being almost unknown to the public is that some non-stigmatised participants seemed to "enjoy", for want of any better word, the 'felt exclusivity' of their condition (and this may be particularly the case in those with *situs inversus*). Although stigmatised and non-stigmatised groups report chronic cough (and symptom scores did not differ – see Table 3), the non-stigmatised group seemed to have assimilated PCD into their personalities, whilst others held PCD more at a distance, allowing a more adverse effect upon their lives, as if the stigmatised group regarded cough as stigmatising, whereas the non-stigmatised group regarded it merely as a nuisance.

### Knowledge of PCD

Public knowledge of PCD is very limited, so that cystic fibrosis was sometimes used to anchor understanding of PCD. The imbalance between the felt seriousness of PCD and public knowledge made some participants feel isolated, a frustration exacerbated by clear, specific, outward signs. Some participants, in an attempt to decrease felt isolation, traded off knowledge imbalance against increased risk of increased stigmatisation when using the better known CF as a knowledge anchor.

### Hiding symptoms

Whilst public lack of awareness of PCD effectively means there is no stereotype, some symptoms are very problematic, making it the symptoms not the disease which is stigmatising [60]. In the questionnaire study [15] a majority of respondents felt embarrassed about coughing in public, a finding consistent with other studies describing the social burden of chronic cough [61,62,62]. The current interviews suggested cough was the most embarrassing feature of PCD, with most participants coughing in private so as not to be overheard. The salience of coughing for stigma is explained by Jones *et al*'s [43] peril dimension, with interviewees suggesting coughing was anti-social and not to be witnessed in public, and some individuals with PCD perceived they may be putting others at risk through coughing. Comments about "smoker's cough" may also refer to Jones *et al*'s dimension of perceived responsibility, a problem that may become more problematic for people with PCD as smoking itself becomes increasingly discredited.

Concealing PCD symptoms also means hiding treatments, in particular postural drainage, with one participant missing treatment to conceal coughing. Concealment may be associated with stress due to fears of being stigmatised [63], a double bind since concealment then worsens the stigmatised illness by avoidance of treatment.

### Mistrust of medical care

Isolation and stigma in some participants was compounded by mistrust in medicine, a mistrust sometimes present from an early age resulting from uncertainty of diagnosis and inappropriate treatment, as also occurs in cystic fibrosis [22]. Despite older participants saying that current medical knowledge of PCD was superior to when they were younger, they nevertheless felt isolation and resentment towards medicine, the sense of isolation being heightened by GPs' unawareness of PCD, and participants feeling merely a "curiosity". Despite some cynicism concerning medicine, some participants undoubtedly had trust in those treating them, a trust which was sometimes life-long.

### Stigma questionnaire

As well as investigating perceptions of PCD, this study was also designed to validate the stigma scale [15]. The perfect correlation of SW's stigma with the previous stigma scores suggests the questionnaire may be a useful in people living with PCD. The questions did focus primarily on information management, and hence felt rather than enacted stigma, and an expanded questionnaire could take other factors into account, particularly those contributing towards isolation.

### Questionnaire validation

Our somewhat unusual method of validation perhaps requires further justification. SW inevitably knew that participants were differentiated on the stigma questionnaire, whose wording he knew (although not of course individual's responses). The questionnaire wording was derived from other stigma measures (providing convergent validity), and corresponded to lay and social science usage of the term 'stigma', giving content validity. Had SW had verbally asked participants those exact questions, then there would be long-term reliability over nearly three years. However the specific wording was explicitly not used (and indeed, despite SW discussing 'stigma' with participants, the word itself is not in the questionnaire), so concordance of questionnaire and the very different method of assessing stigma nearly years later provides long-term predictive validity. Finally, SW's prediction was stigma specific, since not even the five contemporaneous measures of health status in 2003 could significantly distinguish high and low stigma groups (Table 3).

### Limitations

Whilst the concordance of the stigma ratings in 2005 with the stigma scores from 2003 is encouraging, there should also be some caution. SW's stigma ratings were based both on verbal content of interviews and on body language and non-PCD related information. Further work would benefit from stigma ratings based on transcripts alone by individuals uninvolved with interviewing. Although SW the interviewer was blind to the stigma scores, he was aware that participants were matched within pairs, giving the potential problem that if a participant was judged as stigmatised, the other then had to be judged as non-stigmatised. A variant design could blind the interviewer both to stigma scores and the pairings. Fortunately that design can be implemented in a future study using stigma judgements based only on the twelve transcripts.

## Conclusion

Since Goffman's seminal work on stigma [41] there have been numerous attempts to refine and further understand the various elements that underpin stigma, although a full understanding of stigma still remains elusive [64]. Stereotyping and prejudice undoubtedly form part of the process, but embarrassment and shame are also important. PCD is interesting for stigma research as it defies some conentional concepts associated with stigma, as little is known about PCD in the general population, and the outwardly noticeable signs are similar to those of bronchitis or a common cold, and yet our results suggest some people with PCD feel stigmatised and isolated, the barrier of isolation also extending to the medical profession. Problems of uncertainty over diagnosis and perceived lack of understanding by GPs are a worry for some people with PCD, supporting the conclusion of a paper on best practice in care which proposed clearer lines of communication between specialist and GP [4], particularly as some participants reported almost no specialist contact, and hence no specialised monitoring, since initial diagnosis. Some people with PCD may benefit from counselling, especially those who feel isolated and stigmatised by the condition. The ability to assess stigma in people with PCD using a validated questionnaire may help improve support and identify those likely to benefit from counselling.

## Abbreviations

PCD Primary ciliary dyskinesia

GP General practitioner (primary care practioner)

SI *Situs inversus totalis*

SS *Situs solitus*

SW Simon Whalley (author)

ICM I C McManus (author)

## Competing interests

The author(s) declare that they have no competing interests.

## Authors' contributions

The original survey of individuals with PCD was designed, organised and analysed by ICM. The idea for the present study was ICM's, and he was responsible for the selection of participants for the qualitative interview study. SW was responsible for interviewing the participants, and carrying out the grounded theory analysis. The first draft of the manuscript was written by SW, and revision of the manuscript was carried out by both authors.

## Pre-publication history

The pre-publication history for this paper can be accessed here:



## Figures and Tables

**Table 1:** The questions used to assess stigma, which were embedded amongst other questions on respiratory problems and health in general.

**These are questions about other effects that *breathing or respiratory problems *may have. **If you do not have a particular problem then please tick 'Not applicable'.	*Definitely agree*	*Agree somewhat*	*Disagree somewhat*	*Definitely disagree*	*N/A*	*Factor loading*
My coughing or breathing is embarrassing in public	**37.3% **(25/67)	**37.3% **(25/67)	**13.4% **(9/67)	**11.9% **(8/67)	N = 4	*.530*
My breathing problem is a nuisance to my family, friends, or neighbours	**15.6% **(10/64)	**17.2% **(11/64)	**18.8% **(12/64)	**48.4% **(31/64)	N = 7	*.478*
I have sometimes felt I had to hide from other people the fact that I had PCD	**25.4% **(17/67)	**19.4% **(13/67)	**10.4% **(7/67)	**44.8% **(30/67)	N = 4	*.880*
I have sometimes felt embarrassed in public because of having PCD	**21.2% **(14/66)	**28.8% **(19/66)	**12.1% **(8/66)	**37.9% **(25/66)	N = 5	*.818*
I have sometimes avoided situations where people might find out that I had PCD	**23.1% **(15/65)	**13.8% **(9/65)	**6.2% **(4/65)	**56.9% **(37/65)	N = 6	*.863*
I have sometimes felt worried about other people's reaction to PCD	**21.2% **(14/66)	**24.2% **(16/66)	**13.6% **(9/66)	**40.9% **(27/66)	N = 5	*.866*
**How does *PCD *affect your daily life?**	*True*	*False*			*N/A*	*Factor loading*
I sometimes feel it makes people reject me	**18.8% **(13/69)	**81.2% **(56/69)			N = 2	*.586*
Sometimes it makes it more difficult for me to make friends	**16.2% **(11/68)	**83.8% **(57/68)			N = 3	*.592*
It is sometimes difficult telling people about my condition	**43.5% **(30/69)	**56.5% **(39/69)			N = 2	*.860*
Sometimes I prefer not to let other people know that I have PCD	**59.4% **(41/69)	**40.6% **(28/69)			N = 2	*.710*
Sometimes it is difficult having a rare and unusual condition	**56.5% **(39/69)	**43.5% **(30/69)			N = 2	*.622*

The descriptive statistics are taken from our previous paper [15], and show the percentage of individuals giving each of the answers, along with the loading of each measure on the single factor that was extracted in that analysis. NA = "not applicable"

**Table 2:** Demographic details of participants in matched pairs, along with stigma scores from the questionnaire in 2003 and ratings of stigma by the interviewer in 2005.

**Participant code**	**Sex**	**Age at interview**	**Age at diagnosis**	***Situs inversus***	**Stigma score based on questionnaire in 2003 (higher scores indicate higher stigma)**	**Stigma rating of interviewer in 2005. 1 indicates low perceived stigma and 4 indicates high perceived stigma.**
*1A*	*F*	*54*	*32*	*Yes*	*-.46*	*1*
**1B**	**F**	**57**	**23**	**Yes**	**1.61**	**4**
*2A*	*F*	*65*	*58*	*Yes*	*-.37*	*2*
**2B**	**F**	**61**	**50**	**Yes**	**2.05**	**3**
*3A*	*F*	*49*	*34*	*Yes*	*-.08*	*1*
**3B**	**F**	**42**	**22**	**Yes**	**1.91**	**4**
*4B*	*F*	*53*	*43*	*No*	*-.18*	*1*
**4A**	**F**	**51**	**36**	**No**	**1.51**	**4**
*5A*	*F*	*27*	*11*	*No*	*-.87*	*1*
**5B**	**F**	**32**	**10**	**No**	**1.16**	**3**
*7B*	*M*	*42*	*35*	*No*	*-.73*	*1*
**7A**	**M**	**65**	**56**	**No**	**2.05**	**4**

Within each pair the low stigma individual based on the questionnaire is shown in italics and the high stigma individual in bold. It should be noted that individuals were randomly allocated as A or B using random number tables.

**Table 3:** Clinical details of participants in matched pairs, assessed by questionnaire in 2003.

	**St George's Respiratory Questionnaire**			**SF-36 component scores**	
**Participant**	**Symptoms**	**Activity**	**Impact**	**Physical**	**Mental**
*1A*	*54*	*72*	*63*	*47*	*56*
**1B**	**39**	**88**	**69**	**38**	**33**
*2A*	*11*	*88*	*73*	*19*	*52*
**2B**	**32**	**50**	**43**	**26**	**32**
*3A*	*64*	*81*	*77*	*46*	*45*
**3B**	**29**	**81**	**74**	**57**	**28**
*4B*	*11*	*38*	*57*	*32*	*58*
**4A**	**7**	**34**	**42**	**18**	**35**
*5A*	*79*	*59*	*95*	*56*	*41*
**5B**	**39**	**78**	**70**	**42**	**31**
*7B*	*39*	*69*	*98*	*60*	*28*
**7A**	**7**	**78**	**61**	**27**	**47**
					
*High stigma: mean (SD)*	*42.9 (28.0)*	*67.7 (17.8)*	*77.0 (16.4)*	*43.1 (15.3)*	*46.5 (11.2)*
**Low stigma: mean (SD)**	**25.6 (14.9)**	**68.2 (21.0)**	**59.9 (14.3)**	**34.7 (14.0)**	**34.5 (6.8)**
Paired t-test	t (5) = 1.83	t (5) = -.047	t (5) = 2.53	t (5) = 1.30	t (5) = 1.84
	p = .126	p = .964	p = .053	p = .249	p = .125

Within each pair the low stigma individual based on the questionnaire is shown in italics and the high stigma individual in bold.
